# Hepatitis C Virus Infection and Risk of Stroke: A Systematic Review and Meta-Analysis

**DOI:** 10.1371/journal.pone.0081305

**Published:** 2013-11-12

**Authors:** He Huang, Rongyan Kang, Zhendong Zhao

**Affiliations:** MOH Key Laboratory of Systems Biology of Pathogens, Institute of Pathogen Biology, Chinese Academy of Medical Sciences & Peking Union Medical College, Beijing, China; The Chinese University of Hong Kong, Hong Kong

## Abstract

**Background/Aims:**

Several studies analyzed the association between hepatitis C virus (HCV) infection and the risk of stroke or cerebrovascular death, but their findings were inconsistent. Up to date, no systematic review about the association between HCV infection and stroke was performed. We conducted a meta-analysis to examine whether HCV infection dose increase stroke risk in comparison to the population without HCV infection.

**Methods:**

We followed standard guidelines for performance of meta-analysis. Two independent investigators identified eligible studies through structured keyword searches in several databases. Random-effects and fixed-effects models were used to synthesize the data. Heterogeneity between studies and publication bias were also accessed.

**Results:**

Combining the data from the eligible studies, we calculated the pooled multi-factor adjusted Odds Ratio (OR) with 95% confidence interval (CI). Upon the heterogeneity found between studies, the result was 1.58 (0.86, 2.30) by random-effects model. However, after omitting the study which induced heterogeneity, the pooled OR with 95% CI was 1.97 (1.64, 2.30).

**Conclusions:**

This meta-analysis suggested that HCV infection increased the risk of stroke. More prospective cohort studies will be needed to confirm this association with underlying biological mechanisms in the future.

## Introduction

Hepatitis C virus (HCV) has infected more than 170 million people worldwide (approximately 3% of the world’s population) [1]. In up to 80% of infected patients, HCV established persistent infection, often leading to liver cirrhosis and hepatocellular carcinoma [2,3]. Additionally, HCV infection can induce non-hepatic diseases such as type II diabetes [4]. Current therapy is carried out with peg-interferon plus ribavirin, but only a portion of patients can eliminate the virus, and the outcome of treatment is dependent on the viral genotype [5].

Stroke is the second leading cause of death worldwide [6-8]. It is becoming a great health burden in most industrialized countries in future decades. Conventional risk factors for stroke include cardiac diseases, hypertension, diabetes, smoking, alcohol consumption, unhealthy diet, abdominal obesity, lack of exercise, psychosocial stress and depression[8]. Increased evidence indicates that acute and various chronic infectious diseases are important triggers of or risk factors for stroke (reviewed in 9 ). However, the association of HCV infection and stroke is not well established.

Several epidemiological studies have demonstrated that chronic HCV infection is an independent risk factor of stroke or cerebrovascular death [10-15]. However, Younossi et al. did not find an association between HCV and stroke [16]. Until present, there has been no systematic analysis performed yet. We therefore decided to conduct a meta-analysis of published case-control and cohort studies, which permitted to evaluate the role of HCV infection in stroke. In addition to providing a greater understanding about the association between HCV infection and stroke, the finding of the meta-analysis might also give suggestion for future research and help inform clinical practice guidelines.

## Methods

### Eligibility criteria

The published guidelines for the conduct and reporting of meta-analyses were followed [17]. We did not have a registered protocol. All published epidemiologic studies providing an estimate of risk of stroke among HCV infected people compared to normal population or an estimate of risk of HCV infection among people with stroke compared to without stroke were considered in this meta-analysis. 

 Studies were excluded if: the exposed and unexposed groups came from different geographically and temporally defined underlying population; the studies included children, post-transplant recipients, or pregnant women; the studies did not provide risk estimates or data necessary to calculated them; the studies were not published in English. If different overlapping studies used the same study population, only the largest or most recent eligible report was included. 

 Two investigators (He Huang and Rongyan Kang) independently reviewed all identified titles, abstracts and manuscripts to determine if a study was suitable for the meta-analysis. If disagreements appeared, a third investigator will help to resolve the problem.

### Search strategy

To identify all potentially eligible studies, two investigators independently conducted searches in selected databases including Embase, Medline, Conference Proceedings Citation Index-Science (CPCI-S), and Cochrane library. Besides, Google scholar was searched to find additional studies. Searches included the combinations of the keywords “hepatitis c”, “hepatitis c virus”, “hepatitis non A non B”, “HCV”, “stroke”, “cerebrovascular event” and “cerebrovascular disease”. In addition, we reviewed the references from all retrieve articles and relevant reviews to find the studies not captured by the search. Searches were updated as of August 21, 2013. 

### Data abstraction

Two investigators (He Huang and Rongyan Kang) independently extracted data from eligible studies. Data were abstracted and entered into a structured database. The following data were extracted from each study: the first author’s last name, publication year, country where the study conducted, study design, number of subjects, Hazard Ratio (HR), Rate Ratio (RR), or Odds Ratio (OR) with corresponding 95% Confidence interval (95%CI), and confounders adjusted for in multivariate analysis. Meanwhile, the study quality was evaluated using the 9-star Newcastle-Ottawa Scale[18], a validated technique for assessing the quality of non-randomized studies in meta-analysis.

### Statistical analysis

We retrieved unadjusted and adjusted risk estimates presented in original studies. If not available, unadjusted risk estimates will be calculated with crude data extracted from the studies. We examined heterogeneity in results across studies by using *I* ^2^ statistics[19]. The null hypothesis that the studies are homogeneous was rejected if the *p* value for heterogeneity was *p*<0.10 or *I* ^2^>50%. Random-effects model were applied using the DerSimonian-Laird method [20] if heterogeneity was found, otherwise fixed-effects model was used. All meta-analyses were presented as forest plots with risk estimates for all individual studies as well as the overall pooled estimator. The weight of all individual studies was also provided. If heterogeneity was found, sensitive analysis would be performed to indentify potential source of heterogeneity. Publication bias was assessed with Begg’s and Egger’s tests [19,21] and Begg’s funnel plot was presented in this study. The meta-analysis was performed using STATA 10.0 software (StataCorp LP, USA).

## Results

### Searches

**Figure 1:**
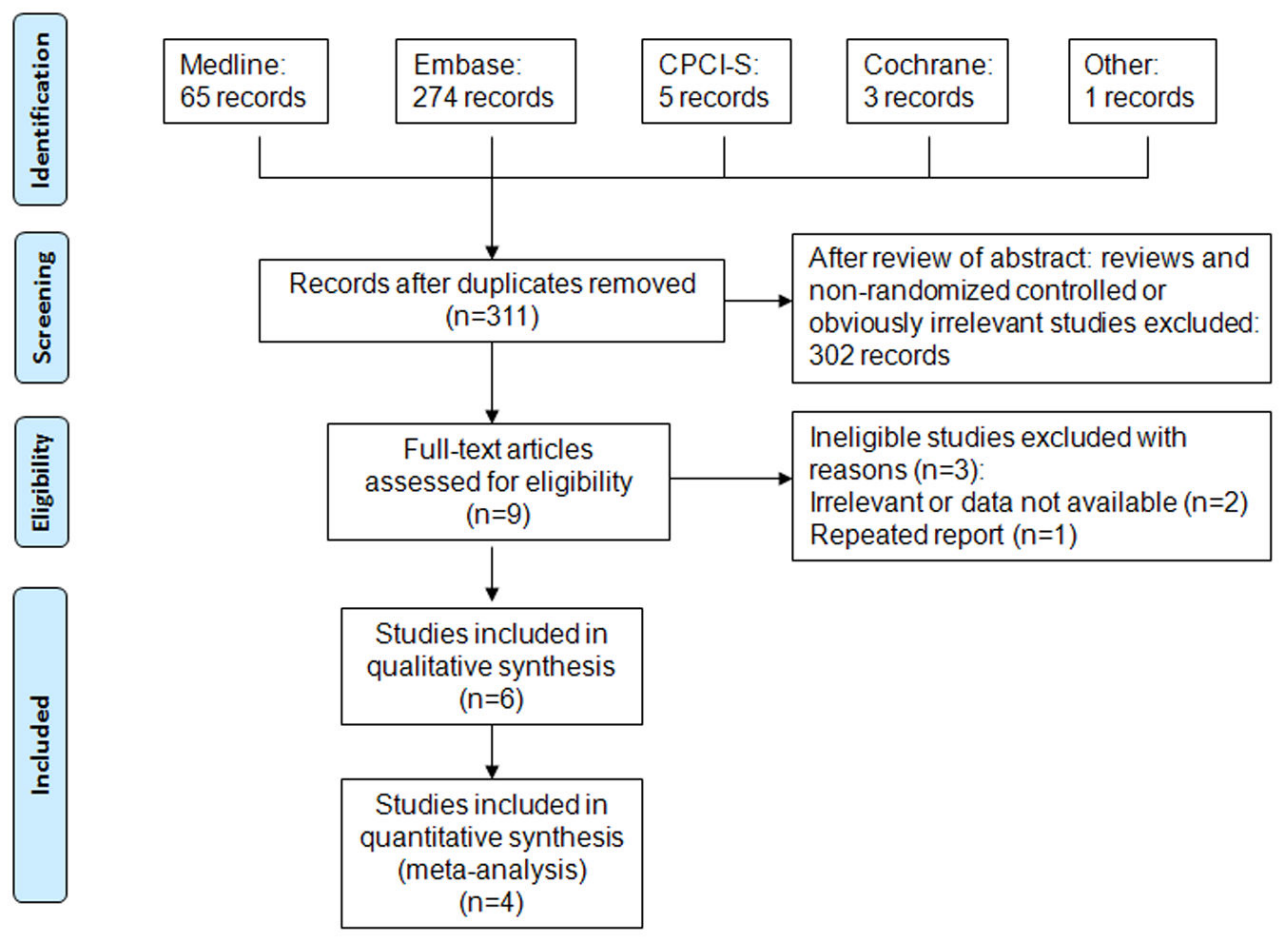
Flow diagram of study identification.

The flow diagram of study identification was shown in Figure 1. The searches retrieved a total of 348 records. After deleting duplicated studies, 311 reports were pooled. After initial review of the abstracts, 9 potentially relevant studies [10-16,22,23] were identified and full-text was retrieved for detailed evaluation. The study, Lee (2010) [11], was excluded because only cerebrovasular death was investigated in this study. Vinikoor (2013) did not provide data on stroke and HCV[22]. Restivo (2013) was a repeated report of Adinolfi (2013) [13,23]. The other 6 studies met the criteria and data was collected [10,12-16].

### Study characteristics

The main characteristics of the six identified studies were shown in Table 1 and Table 2. Of the six studies, five were retrospective studies and one was prospective study. Two studies reported HRs with 95% CI, three studies reported ORs with 95% CI, and the other one reported RR with 95% CI. Five studies were population-based studies and the other one was performed in hospital. All studies were conducted in developed areas.

**Table 1:** The main characteristics of identified studies reporting Odds Ratio or Rate Ratio.

Ref (#)	Study ID (Year)	Country/ territory	Study timing (Measure of association)	Source	Case (n)	Control (n)	HCV positivity criteria	Unadjusted risk estimates (95% CI)	Adjusted risk estimates (95% CI)
13	Adinolfi (2013)	Italy	Retrospective (OR)	Hospital	HCV+ (79)	HCV- (741)	anti-HCV ELISA	1.81 (1.51, 2.16)	2.04 (1.69, 2.46)*
10	Forssen (2009)	U.S.	Retrospective (RR)	Population health plan	HCV+ (21919)	HCV- (67109)	ICD-9 codes 070.44, 070.54, 070.7, 070.70, and 070.71	NA	1.76 (1.23, 2.52)**
14	Gutierrez (2012)	U.S.	Retrospective (OR)	NHANES (2005-2010)	HCV+ (NA)	HCV- (NA)	NA	NA	9.61 (2.58, 35.78)***
16	Younossi (2013)	U.S.	Retrospective (OR)	NHANES (1999-2010)	HCV+ (173)	HCV- (19568)	HCV RNA PCR	NA	0.58 (0.16, 2.02)****

NHANES: National Health and Nutrition Examination Surveys, NA: not available.

* Adjusted for age, gender, hypertension, smoking, diabetes, hyperlipidemia, past ischemic heart events, and artial fibrillation.

** Adjusted for age, gender, hypertension, and steroid use.

*** Adjusted for demographics and cardiovascular risks (not specified).

**** Adjusted for age, smoking, and diabetes.

**Table 2:** The main characteristics of identified studies reporting Hazard Ratio.

Ref (#)	Study ID (Year)	Country/ territory	Study timing (Measure of association)	Source	Case (n)	Control (n)	HCV positivity criteria	Unadjusted risk estimates (95% CI)	Adjusted risk estimates (95% CI)
12	Hsu (2013)	Taiwan	Retrospective (HR)	LHID2000	HCV+ (3113)	HCV- (12452)	ICD-9-CM 070.41, 070.44, 070.51, 070.54, and V02.62	NA	1.23 (1.06, 1.42)*
15	Liao (2012)	Taiwan	Prospective (HR)	TNHIRD	HCV+ (4094)	HCV- (16376)	ICD-9-CM 070.41, 070.44, 070.51, and 070.54	1.30 (1.17, 1.44)	1.22 (1.13, 1.40)**

TNHIRD: Taiwan National Health Insurance Research Database, LHID2000: Longitudinal Health Insurance Database 2000, NA: not available.

* Adjusted for age, gender, hyperlipidaemia, diabetes, heart disease, hypertension, alcohol-related illness, chronic obstructive pulmonary diseases, aspirin use, clopidogrel use, warfarin use, dipyridamole use, ticlopidine use, statin use, ACE inhibitors use, and influenza vaccination.

** Adjusted for age, gender, hypertension, smoking, diabetes, hyperlipidemia, heart disease, and drug use.

### Meta-analysis

Because stroke was a relatively rare outcome in the population (<3%) studied in Forssen (2009) [10], the distinctions between RR and OR could be ignored [24]. Therefore, the reported RR could be seen as OR and combined with ORs reported by other three studies [13,14,16]. However, the other two studies reporting HRs could not be included in the analysis [12,15].

**Figure 2:**
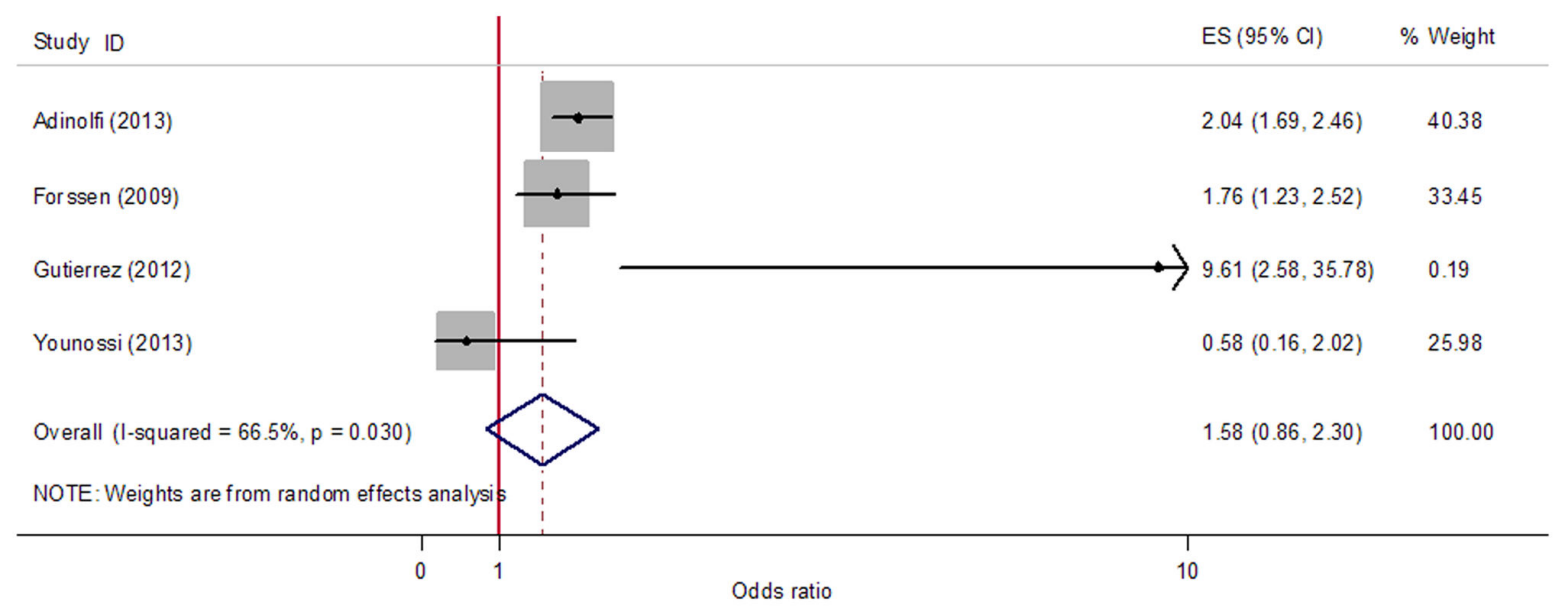
Forest plot for meta-analysis comparing risk of stroke in HCV infected patients compared to that in non-infected controls. Four studies reporting ORs and RR were included. Adjusted ORs from included studies and the pooled OR was shown. Dimension of shaded OR for individual studies is proportional to their total weight in calculation of the pooled estimator.

**Figure 3:**
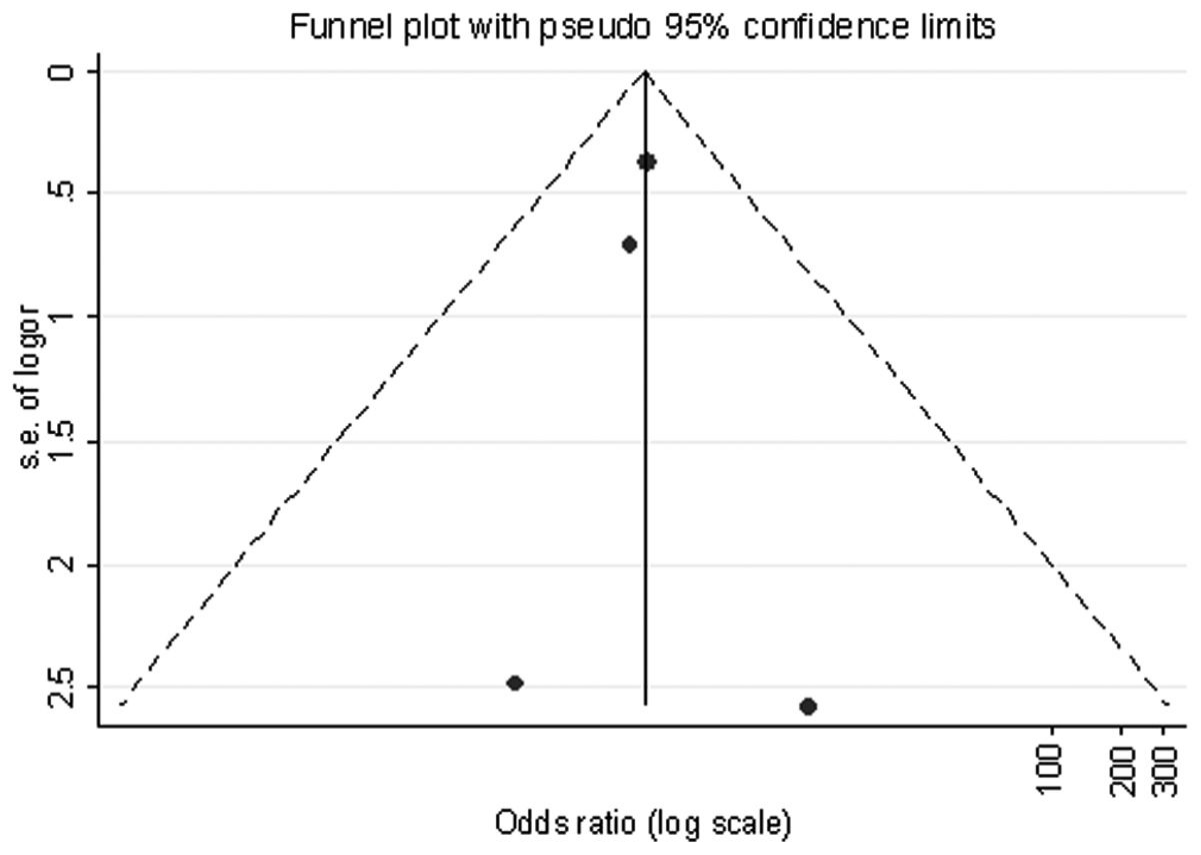
Begg’s funnel plot (with pseudo 95% confidence intervals) to detect any publication bias.

Because heterogeneity was found between the four included studies (*p*=0.030, *I* ^2^=66.5%), random-effects model was used to calculate the pooled risk estimator. By combining data form the four studies, the pooled OR (95% CI) for stroke risk with HCV was 1.58 (0.86, 2.30) (Figure 2). Publication bias was not suggested by Egger’s test (*p*=0.978) and Begg’s funnel plot (Figure 3).

Because of the limited number of studies reporting HRs, meta-analysis could not be performed to estimate the pooled HR. 

### Sources of heterogeneity and sensitive analysis

**Figure 4:**
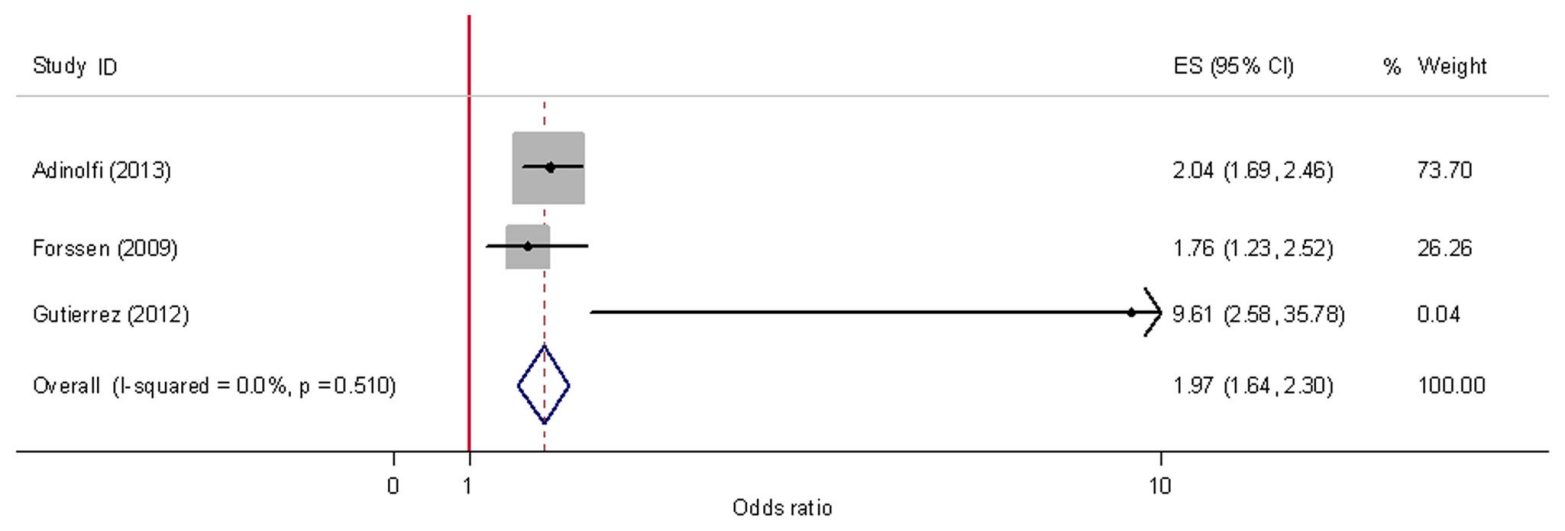
Forest plot for meta-analysis comparing risk of stroke in HCV infected patients compared to that in non-infected controls. After omitting the study which induced heterogeneity, adjusted ORs from the other three studies and the pooled OR was shown. Dimension of shaded OR for individual studies is proportional to their total weight in calculation of the pooled estimator.

Because the inclusion of studies which induce heterogeneity may causes bias to the final conclusion, we decided to perform the sensitive analysis to explore the heterogeneity among studies. A sensitivity analysis omitting one study at a time and calculating the pooled ORs for the remainder of the studies showed that the study by Younossi et al. might be the key contributor to the between-study heterogeneity. No heterogeneity was observed after excluding this study (*p*=0.510, *I* ^2^=0.00%) as shown in Figure 4. The pooled OR (95% CI) for stroke risk with HCV was 1.97 (1.64, 2.30). Publication bias was not suggest by Egger’s test (*p*=0.491) and Begg’s funnel plot (not shown).

## Discussion

As we know that the result of a single research may be affected by many factors, in order to reduce the bias and increase the efficiency of the small sample study of statistics, meta-analysis was performed to further explore the relationship between stroke and HCV infection. Six studies estimated the risk of stroke in HCV infected population were identified [10,12-16]. The final analysis suggested that HCV infection increased the risk of stroke with statistical significance. To our knowledge, this was the first to attempt to synthesize the existing world literature to evaluate the effect of HCV infection on stroke.

The mechanism(s) by which HCV may favor stroke is not known. Increasing evidence has showed that chronic HCV infection increased the risk of ultrasonographically defined carotid intima-media thickness and or plaque [25-27], which are predictors of cerebrovascular disease [28]. It is well-known that chronic inflammation plays an important role in the instability of plaque [29]. It was also found that HCV replicated with carotid plaque [30]. Beyond, HCV infection also increased the risk of metabolism diseases, such as type II diabetes[4]. All of these events potentially increased the risk of stroke. Thus, it is believed that there is potential association between HCV infection and stroke. However, studies from several groups provided conflicting results. Our meta-analysis consisted of more than 22171 HCV infected individuals and more than 87418 controls and might allow a much greater possibility of reaching reasonably strong conclusions. 

The results of combing the four eligible studies suggested that HCV infection was not associated with stroke (Figure 2). However, a substantial heterogeneity among the studies included was found, which can influence the validity and reliability of the results of meta-analysis. Thus, sensitivity analysis was performed to identify the potential sources of between-study heterogeneity and to reduce heterogeneity [31]. Our analyses found that the study by Younossi et al. [16] was a major contributor to the heterogeneity. Inadequate adjusting variables used might induce bias and could be an important source of heterogeneity. We noted that several cofounders, such as race, gender, and hypertension, were significantly different between HCV+ and control in this study [16]. However, these factors were not involved in the adjustment. The differences in HCV positive criteria used might be another source of heterogeneity. After omitting this study, heterogeneity was reduced and the results suggested that HCV infection significantly increased the risk of stroke (Figure 4).

Some limitation in this meta-analysis should be demonstrated in the discussion of the results. Firstly, our search was limited to studies published in English. However, we found no evidence of publication bias, although the statistical tests for detecting this had limited power[32], especially for relatively small numbers of studies. Secondly, we should noted that the number of included studies in this analysis was relatively low (4 studies). Two of the studies were published only in abstract form on conferences[10,14] and the study by Adinolfi et al.[13] was the only one published article in the final analysis (Figure 4). This limitation might induce bias, although publication bias was not found in our analysis. Thirdly, HCV positive criteria and cofounder for adjusting ORs were different between the included studies, which might induce bias in our study. Fourthly, all the included studies were retrospective studies and the inherent limitations of such studies may influence our findings. More studies with prospective design will be needed. Fifthly, the studies were restricted to United States and Italy, so it was uncertain whether these results were generalizable to other populations. The other two studies excluded in the final analysis were performed in Taiwan [12,15]. However, they reported HRs which could not be combined with ORs. Never the less, the results form these two studies both supported our conclusion. Due to the limitations mentioned above, the results of this meta-analysis should be interpreted with care.

In conclusion, our meta-analysis revealed that  HCV infection significantly increased the risk of stroke. Due to the limitations mentioned above, more population-based well-designed cohort studies will be needed to confirm our results. Furthermore, future studies may evaluate the impacts of different genotypes of HCV infection on stroke. The updating of this meta-analysis will give us more information and may help inform clinical practice guidelines in the future.

## Supporting Information

Checklist S1 **PRISMA Checklist for this meta-analysis.**
(DOC)
